# Transvaginal laparoscopic salpingo-oophorectomy: an oncological risk-reducing procedure

**DOI:** 10.2144/fsoa-2019-0089

**Published:** 2019-11-05

**Authors:** Kostas Lathouras, Srdjan Saso, Benjamin P Jones, Sarah Bowden, Maria Kyrgiou, Anna Stienen-Durand, Gareth Beynon

**Affiliations:** 1 Imperial College Healthcare NHS Foundation Trust, Queen Charlotte's and Hammersmith Hospitals, Du Cane Road, London W12 0HS, United Kingdom; 2 Frimley Health NHS Foundation Trust, Portsmouth Road, Frimley, Camberley GU16 7UJ, United Kingdom

**Keywords:** *BRCA1/2*, natural orifice transluminal endoscopic surgery, salpingo-oophorectomy, transvaginal endoscopy

## Abstract

**Aim:**

Since the first natural orifice transluminal endoscopic surgery procedure, renewed interest has arisen in further developing and advancing minimal access surgery. We introduce a natural orifice endoscopic approach for a bilateral salpingo-oophorectomy.

**Patients & methods:**

Using the vagina as a natural orifice, we performed a transvaginal laparoscopic salpingo-oophorectomy to remove bilateral adnexa in patients with a strong family history of ovarian and/or breast cancer and those positive for *BRCA1/2* mutation.

**Results:**

Total 36 women underwent transvaginal laparoscopic salpingo-oophorectomy. Conversion to routine laparoscopy was required in eight patients to complete the operation. No peri-operative complications were noted.

**Conclusion:**

We describe a novel approach in gynecological surgery. Our technique proved to be safe and efficient with the advantage of avoiding any abdominal scars.

## Lay abstract

This article describes a novel, keyhole surgical approach to remove tubes and ovaries in a female patient. The approach is known as a ‘natural orifice endoscopic approach’ and involves using the vagina as a natural orifice. Both tubes and ovaries were removed in patients with a strong family history of ovarian and/or breast cancer and those positive for *BRCA1/2* genetic mutation. Two ports are inserted into the vagina to access the abdomen with both specimens subsequently retrieved vaginally. Total 36 women underwent the procedure with no complications. The technique proved to be safe and efficient with the advantage of avoiding any abdominal scars compared with routine keyhole surgery.

Indications for bilateral salpingo-oophorectomy (BSO) in the context of ovarian pathology are well established. However, indications for BSO in the absence of ovarian pathology are less recognized. BSO as a part of planned treatment for hormone sensitive breast cancer has been shown to be of benefit [1,2]. For patients with the *BRCA1* mutation, the cumulative risk of ovarian cancer at 70 years of age is 39% [3]. Hence, prophylactic BSO in patients with *BRCA1* or *BRCA2* mutation has been shown to reduce ovarian cancer risk by 96% [4]. It has also been demonstrated to reduce the risk of breast cancer by 25–35% [5]. The procedure has therefore been recommended as a risk-reducing technique for patients of 40 years of age or over [6].

Minimally invasive techniques result in reduced recovery time making prophylactic BSO more acceptable to those patients with *BRCA1* and *BRCA2* mutations. The development of minimally invasive techniques for prophylactic BSO has, to date, been focused on mainly laparoscopic techniques. Vaginal techniques include endoscopic salpingo-oophorectomy (SO) but only when combined with vaginal hysterectomy [7,8].

Natural orifice transluminal endoscopic surgery (NOTES), first developed by a multicenter team of investigators (the Apollo Group) in the late 1990s, is an emerging field which is evolving rapidly. It allows for access to the peritoneal cavity via a hollow viscus and thus, for diagnostic and therapeutic procedures to be performed [9,10]. NOTES have been performed through transgastric initially, and subsequently transcolonic, transvaginal and transurethral/transcystic approaches [11–14].

In this manuscript, we describe and introduce a NOTES for a BSO, whereby the removal of the fallopian tubes and ovaries is through the body surface of the vaginal natural orifice.

## Patients & methods

A total of 36 patients were recruited and managed with laparoscopic transvaginal BSO from January 2010 to September 2015 (Frimley Park Hospital, Surrey, UK). Ethical approval was not required. Indications for this procedure were previous history of breast cancer, familial history of breast and/or ovarian cancer,* BRCA1/BRCA2* gene mutations, symptomatic ovarian cysts and severe premenstrual syndrome. Patient characteristics are presented in Table 1.

**Table 1.** Patient characteristics.

Patient characteristics	Patient number (n = 36)	Further information
Age (years)		Median 52 (35–68)
BMI		Median 25.4 (19–36)
Indication: – *BRCA1/2* – Family history of breast/ovarian cancer – History of breast cancer – Benign ovarian cyst – Severe premenstrual syndrome	7 14 2 3 2	
Completed successfully: – Yes – No	28 8	All eight converted to conventional laparoscopy
Length of stay: – Day 0 – Day 1	14 14	
Estimated blood loss		0 (one case: EBL 50 ml)
Complications – Grade 1 Dindo	3	Temporary urinary retention requiring catheterization
Operating time in minutes		Median: 60 min (34–95)

EBL: Estimated blood loss.

Women with a known history of severe pelvic inflammatory disease, posterior vaginal wall procedures, diverticulitis, any laparotomy or laparoscopy involving the sigmoid or the rectum and severe endometriosis were not considered eligible for the procedure. All women underwent a transvaginal ultrasound scan and only patients with normal findings were included in the study.

**Figure 1.**
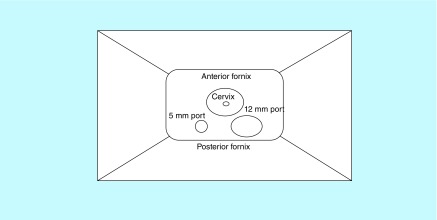
Pictorial representation of port sites in vaginal orifice.

The first laparoscopic transvaginal BSO was performed in 2010 following standard pre-operative assessment and admission on the day of surgery. The technique is described in detail below. Routine vaginal decontamination was performed with 0.05% aqueous chlorhexidine solution at the commencement of surgery. The bladder was emptied via a Foley’s catheter. An initial 30–40 degrees Trendelenburg position was used, allowing for good displacement of both the small and large bowel to minimize the risk of bowel injury. Following instrumentation of the uterus with a Spackman manipulator and anterior version of the uterus, a trocar measuring 15 cm in length and 5 mm in diameter was inserted 1 cm to the right of the midline of the posterior fornix. An acute angle was applied to avoid injury to the rectum. The trocar was held in the left hand of the surgeon, while the uterus was anteverted, with the Spackman manipulator held in the right hand. This initial trocar should not be placed in a more lateral position to avoid injury to the pelvic wall or the right ureter. Following insertion of this primary trocar, a 5 mm rigid camera was inserted to ensure intraperitoneal trocar placement. When this was confirmed, a pneumoperitoneum was developed, with the intra-abdominal pressure set at 12 mmHg. The second trocar (15 cm long and 12 mm in diameter) was inserted 1–2 cm to the left of the initial trocar (Figure 1). At this point, the initial steep Trendelenburg position may be reduced to approximately 25–30 degrees. Prior to commencing the BSO, both ureters are identified in the right and left pelvic side walls. In addition, washings of the peritoneal cavity can be performed at this stage if indicated with routine irrigation and suction.

**Figure 2.**
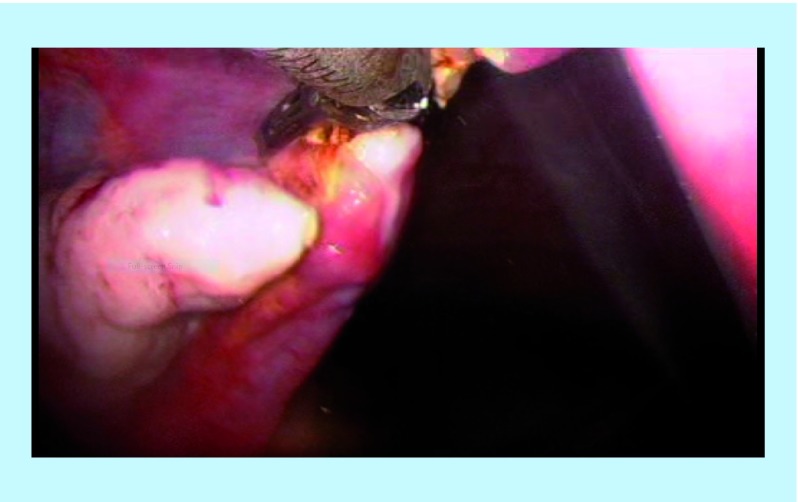
Right utero-ovarian ligament.

**Figure 3.**
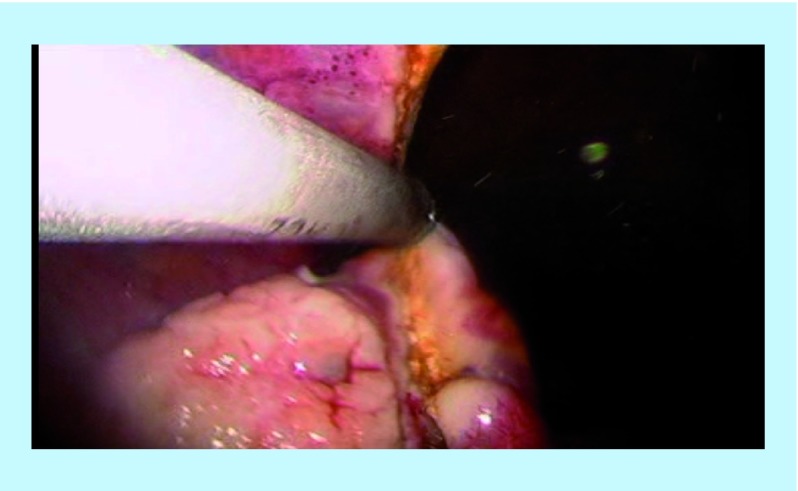
Right infundibulo-pelvic ligament.

The right SO was performed using the Harmonic scalpel inserted through the right 5 mm port. The camera was held within the left port. The assistant continued to hold and manipulate the uterus accordingly with the Spackman manipulator throughout the whole procedure. The dissection began by cutting the ovarian ligament (utero-ovarian ligament) as well as the insertion of the fallopian tube into the uterus (Figure 2). Following that, the broad ligament was dissected (right mesosalpinx) and the right infundibulo-pelvic ligament (IP) was divided (Figure 3). The adnexa is carefully placed at the sacrum promontory or the pouch of Douglass.

**Figure 4.**
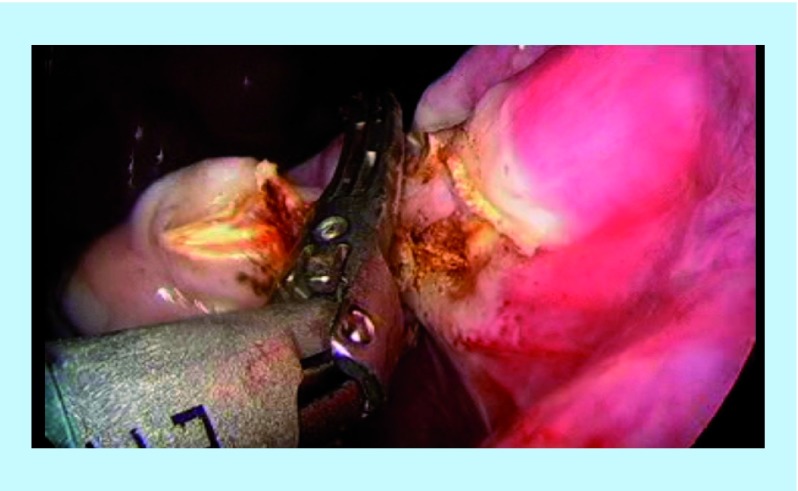
Left utero-ovarian ligament.

**Figure 5.**
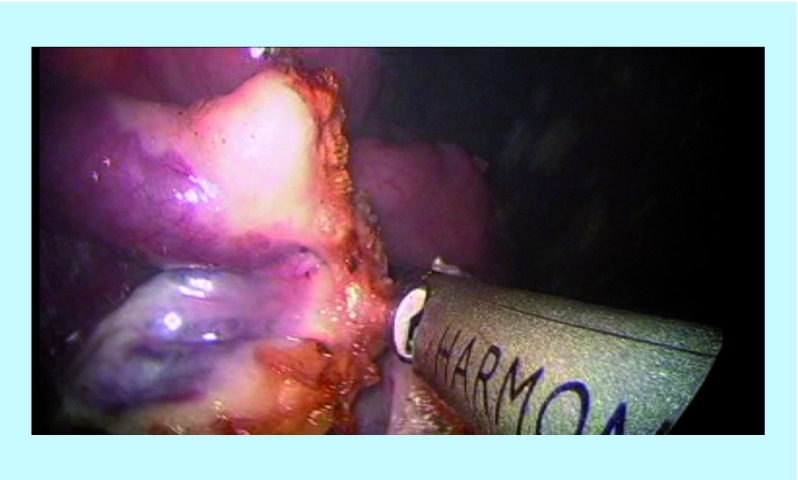
Left infundibulo-pelvic ligament.

The left SO is performed by applying the same steps as above. However, in this case, the Harmonic scalpel might need to be placed within the left port and the camera within the right port. This is to allow easy access to the anatomical position of the left adnexa. The dissection on the left side follows the exact same steps as the dissection on the right. First, the left utero-ovarian ligament was divided (Figure 4), followed by the mesosalpinx of the left fallopian tube. Finally, the left IP ligament was divided (Figure 5). In some patients, the adhesions of the sigmoid colon with the peritoneum of the left pelvic side wall may need to be dissected before achieving access to a safe and efficient division of the left IP. Both specimens are retrieved through the 12 mm port. If the specimens are large in size, the port is taken out and the adnexa are removed through the 12 mm colpotomy. Before completing the procedure, both ureters are inspected to ensure that there is no injury, hemostasis is confirmed and both ports are retrieved. 2/0 vicryl rapid is used to close the small vaginal orifices.

## Results

An overall of 36 patients were considered eligible for the new technique (aged 38–56 years). Total 28 were successfully operated on by applying transvaginal laparoscopic salpingo-oophorectomy (TLSO). Of the eight unsuccessful cases, four were because of a failure to maintain a pneumoperitoneum. One patient had a small left ovarian cyst adherent to the sigmoid colon. In another case, a large number of epiploic appendicae prevented appropriate visualization and safe insertion of the secondary port. Bleeding from the IP ligament and difficulty visualizing the ovarian ligament prevented the success of a further two TLSOs. All of the eight unsuccessful cases were completed by conversion to conventional laparoscopy and the ovaries were retrieved via the posterior colpotomy already performed.

Of the 28 successful cases, operating time varied between 34 and 95 min with a mean time of 60 min. No blood loss was documented in 35 cases while one case had an estimated blood loss of 50 ml. Intra-operative complications were encountered in this particular cohort. These included temporary poor visualization because of bleeding from the pelvic side wall (n = 2). Manipulation of vaginal ports led to a vaginal tear in between them that subsequently caused the loss of pneumoperitoneum (n = 2). Nevertheless, the procedure was completed successfully. In addition, the operation became more complex in seven cases, where adhesions associated with the sigmoid colon either prevented good visualization of the left IP or caused persistent folds of small bowel within the pelvis.

Postoperative morbidity was minor and included only Clavien–Dindo Grade 1 complications with no evidence of pelvic abscess or infection. Three of the patients developed temporary urinary retention requiring catheterization postoperatively, one patient presented with shoulder tip pain and another with abdominal distension and constipation postoperatively.

A total of 14 patients were discharged on the day of their operation and the remaining 14 were fit for discharge on day one. Histology obtained in all cases showed no evidence of ovarian neoplasia. No patients required follow-up as a result of their procedure.

## Discussion

### Main finding

We present our experience in developing and implementing a new surgical technique for laparoscopic BSO using the vaginal natural orifice. We have successfully completed this operation in 28 patients. Hence, we believe that this new method can replace the traditional laparoscopy as it avoids unnecessary abdominal wounds in carefully selected patients.

### Interpretation

This may be a first such report on the transvaginal method of removing tubes and ovaries for oncological reasons. However, the transvaginal approach in removing other gynecological pathologies has already been described a number of times in literature. Examples include the transvaginal approach to ectopic pregnancy [15], the use of transvaginal NOTES to perform an adnexectomy for benign pathology [16] and the potential of transvaginal hydrolaparoscopy as an outpatient procedure for infertility investigation [17].

Our series have demonstrated the safety and feasibility of TLSO. Previously raised concerns about bacterial contamination with the transvaginal approach in data from abdominal and pelvic surgery appear to be unfounded as no patient in our series developed postoperative infection. Evidence suggests that the transvaginal route has lower infection rate compared with the transgastric route initially favored by surgical colleagues [18]. In addition, as shown in fertility patients that require egg retrieval through the vagina, bacterial contamination does not necessarily lead to infection [19].

We believe that the advantage of performing an operation that will spare the patient abdominal scars is much greater than the small risk of conversion to routine laparoscopy. Certain attention should always be given to the placement of the excised specimens before their retrieval. We tend to remove each specimen upon its removal from the 12 mm port. When the size of the specimen does not allow direct retrieval, however, it should be placed temporarily in the peritoneal cavity to avoid unnecessary manipulation and a possible vaginal tear. This is so as to avoid loss of pneumoperitoneum and an adequate visual field. Because of the steep Trendelenburg, this task might be technically difficult and demands attention. We usually place the specimen in the sacrum promontory or the Pouch of Douglas. However, these two placement areas can be risky, as the former can lead to misplacement of the specimen in the upper abdomen, and the latter can obscure vision and compromise removal of the contralateral ovary. Our opinion is that the surgeon should choose wisely where to place the specimen depending on anatomy and body habitus.

All intra- and post-operative factors demonstrate the feasibility, safety and efficiency of TLSO. Blood loss, peri- and post-operative morbidity were minimal and all women were ready to go home a few hours after the completion of the procedure. Half of our cohort stayed overnight either for social reasons or because of the late operative time. We believe that in all above parameters TLSO is at least comparable to routine laparoscopy, even though a direct comparison has not been performed.

We did manage to complete all the operations, with the advantage of new energy laparoscopic devices. For our operations, we applied the Harmonic scalpel, as that was the energy device that our organization provides us (no conflict of interest). The operation technique that we describe here may be potentially performed by any novel laparoscopic advanced-energy device.

This study did not compute the cost of the actual procedures. In general, all procedures were carried out using the same instruments as we do in a conventional laparoscopy to perform BSO. However, instead of using three or four ports, we inserted two ports. In addition, we applied advance-energy devises instead of conventional bipolar and monopolar energy. In theory, when using an advance-energy device, the cost of a procedure is increased. However, in our opinion, the cost of this is offset by reduction in pain, number of complications and days in hospital stay. This was demonstrated in our study.

It is important to note that retrieving nongynecological organs through the vagina is a recognized, low-cost procedure over the last decade. It has been used for common gastrointestinal operations such as cholecystectomy, appendicectomy, sleeve gastrectomy and colon resections. It demands minimal setup and highly trained expertise. Furthermore, transvaginal NOTES is the most common, the safest and is widely accepted because vaginal entry is far less intricate than any other NOTES entry [20].

### Limitations

There are certain limitations with this new technique that surgeons need to be aware of. First, the use of only one instrument (‘one-handed surgeon’) to dissect and divide tissues makes the operation particularly demanding. This becomes even more enhanced when it comes to the dissection and division of the left IP. In this anatomical region, normal adhesions between the sigmoid colon and the left pelvic side wall peritoneum might prevent proper dissection and division leading to bleeding from the IP. For that reason, four of our cases were abandoned and converted to routine laparoscopy. However, in some other cases, these adhesions were successfully dissected and the IP ligament was securely divided.

Two of our cases were also converted to routine laparoscopy because of the inability to maintain the pneumoperitoneum. A vaginal tear connecting the two ports was sustained during manipulation and movement of the ports. For that reason, we recommend a 1–2 cm distance between the placement of the two ports to avoid this particular complication and may be reduce the conversion rate of the procedure. Eight out of the 36 operations were converted to and completed with a routine laparoscopic BSO. However, we have to emphasize that most of our conversions were performed early in the accumulation of our cases and coincided with the learning curve of the procedure. Nonetheless, we believe that even if the procedure is abandoned for any reason described above, the disadvantage for the patient is very small as all cases were easily salvaged with routine laparoscopy.

We believe that with further experience and likely technical developments in the available equipment, the transvaginal approach to oophorectomy has the potential to supersede the currently popular abdominal approach to carefully selected patients. This is mainly because of both the rapid recovery and its cosmetic benefits. Following the success of TLSO, we are now adapting this technique and have already performed one successful vaginal sterilization as a day case procedure with an operating time of 16 min. No complications were noted.

We have to emphasize that TLSO is a demanding operation as described above and the learning curve of the procedure might be longer than in routine laparoscopy. Nevertheless, the advantage of scar sparing is also quite significant and, with proper training, TLSO can replace standard laparoscopy in future women in need of the operation.

## Conclusion

We describe a novel approach in gynecological surgery, the first series of TLSO. Our technique proved to be safe and efficient with the advantage of avoiding any abdominal scars compared with routine laparoscopy. We believe that TLSO, even though surgically demanding, can replace routine laparoscopy in a highly and carefully selected population.

## Future perspective

Interestingly, BSO may not be acceptable to younger women in view of cosmetic reasons yet the transvaginal approach presented herein may not be indicated in younger women. Therefore, we cautiously suggest limiting the transvaginal approach to prophylactic BSO for patients with *BRCA* mutations. This may make the advantages of transvaginal approach more attractive.

**Executive summary**

- Since the first natural orifice transluminal endoscopic surgery procedure, renewed interest has arisen in further developing and advancing minimal access surgery.
- We introduce a natural orifice endoscopic approach for a bilateral salpingo-oophorectomy.
- Using the vagina as a natural orifice, we performed a transvaginal laparoscopic salpingo-oophorectomy (TLSO) to remove bilateral adnexa in patients with a strong family history of ovarian and/or breast cancer and those positive for *BRCA1/2* mutation.
- The technique is based on inserting a 5 mm port in the posterior vaginal fornix and subsequently developing a pneumoperitoneum. Following that, a second (12 mm) port is placed adjacent to the first one and the procedure is performed with the use of a Harmonic scalpel.
- Both specimens are retrieved vaginally, and the port sites are sutured with a vicryl stitch.
- Total 36 women underwent TLSO between January 2010 and October 2015. The procedure was successful in 28 patients. No peri-operative complications were noted. All patients went home either on the day of the operation or the day after.
- We describe a novel approach in gynecological surgery, the first series of TLSO. We believe that TLSO, even though surgically demanding, can replace routine laparoscopy in a highly and carefully selected population.
